# Conservation strategies to mitigate impacts from climate change in Amazonia

**DOI:** 10.1098/rstb.2007.0018

**Published:** 2008-02-11

**Authors:** Timothy J Killeen, Luis A Solórzano

**Affiliations:** 1 Conservation International, Center for Applied Biodiversity Science2011 Crystal Drive, Suite 500, Arlington, VA 22202, USA; 2 Museo Noel KempffCasilla 2458, Santa Cruz, Bolivia; 3 Gordon and Betty Moore FoundationPresidio of San Francisco, PO Box 29910, San Francisco, CA 94129-0910, USA

**Keywords:** conservation corridors, biodiversity, protected area design

## Abstract

Protected area systems and conservation corridors can help mitigate the impacts of climate change on Amazonian biodiversity. We propose conservation design criteria that will help species survive *in situ* or adjust range distributions in response to increased drought. The first priority is to protect the western Amazon, identified as the ‘Core Amazon’, due to stable rainfall regimes and macro-ecological phenomena that have led to the evolution of high levels of biodiversity. Ecotones can buffer the impact from climate change because populations are genetically adapted to climate extremes, particularly seasonality, because high levels of habitat diversity are associated with edaphic variability. Future climatic tension zones should be surveyed for geomorphological features that capture rain or conserve soil moisture to identify potential refugia for humid forest species. Conservation corridors should span environmental gradients to ensure that species can shift range distributions. Riparian corridors provide protection to both terrestrial and aquatic ecosystems. Multiple potential altitudinal corridors exist in the Andes, but natural and anthropogenic bottlenecks will constrain the ability of species to shift their ranges and adapt to climate change. Planned infrastructure investments are a serious threat to the potential to consolidate corridors over the short and medium term.

## 1. Introduction

It is essential that conservation and development strategies for the Amazon reflect its importance as a repository of biodiversity and provider of globally important ecosystem services. Errors in foreseeing the nature of climate change in the Amazon will reverberate across continents and societies.

Climatologists evaluate the consequences of global warming using global circulation models (GCM) and link them to regional models that predict how energy flows will impact regional and local precipitation regimes. Although the models can be highly hypothetical, they are useful in identifying areas at greater or lesser risk to climate change. All the models predict increased warming in the Amazon, but vary regarding future precipitation regimes ranging from increasing wetness to pronounced drought (Li *et al*. 2006). One GCM (HadCM3LC) incorporates principles of plant physiology into its land surface component and shows how increasing temperatures and dryness in the Amazon will increase soil respiration and reduce photosynthesis (Betts *et al*. 2004). The HadMC3LC model predicts that global climate change will initiate a feedback cycle that shifts most of the Amazon from an evergreen forest to a savannah ecosystem within the next century (Betts *et al*. 2004). Analogues for this future scenario are provided by current phenomena related to sea surface temperatures (SST): the positive phase of the El Niño Southern Oscillation with increased SST in the eastern Pacific is associated with depressed wet season rainfall in northern and eastern Amazonia, while a separate dry season phenomenon links reduced rainfall to a tropical Atlantic SST gradient which weakens the trade winds that export water from the Atlantic to the Amazon (Giannini *et al*. 2001).

In spite of these pessimistic predictions, the future precipitation regime of the Amazon is still best described as uncertain. Uncertainty is dealt with in business by adopting a risk management strategy that assesses and monitors risk, while taking action to reduce risk via avoidance, mitigation or adaptation. In the case of the Amazon, multiple scientific efforts are assessing risk and will monitor the impact of climate change (see other papers in this special edition). Mechanisms under consideration by the signatories to the United Nations Framework Convention on Climate Change seeking to compensate countries for reducing emissions from deforestation are examples of avoidance, while efforts to promote sustainable development will help local societies adapt to climate change. In this paper, we show how the large-scale design of protected area systems can help to mitigate the impacts of climate change on biodiversity by identifying potential refugia and incorporating these into corridors that will allow species to persist, shift ranges and ultimately adapt to climate change. Many of the steps necessary to develop a conservation strategy that addresses climate change are also components of traditional conservation strategies and would benefit conservation regardless of climate change.

## 2. Data sources and working hypotheses

Our analyses are based on well-known principles of physical geography, when considered at the continental scale, and fundamental precepts of community ecology, when considered at the local scale. Our goal is to identify regions with inherently stable climates and landscapes in current (or potential) climatic tension zones with geomorphological features that will enhance species persistence in spite of climate change. We base our analyses on the following criteria.

- The greatest threat to lowland ecosystems will come from reduced precipitation and increased seasonality that would lead to a large-scale change in the function of the humid forest ecosystem.
- Air rises adiabatically over higher topographic features causing precipitation to occur at rates and periodicities greater than surrounding areas.
- Soil gradients (texture and depth) constrain plant species distributions due to water retention capacity; consequently, some habitats will ameliorate drought and others will intensify drought.
- Latitudinal and altitudinal gradients are compounded by other multiple environmental factors, including temperature, precipitation, geological substrate, fertility, disturbance, slope, aspect, etc.
- Biomes are the manifestations of multiple species with similar biological traits and geographical distribution patterns and will be reconfigured as the responses of species will alter community composition on landscapes as species are eliminated, added or retained.

**Figure 1:**
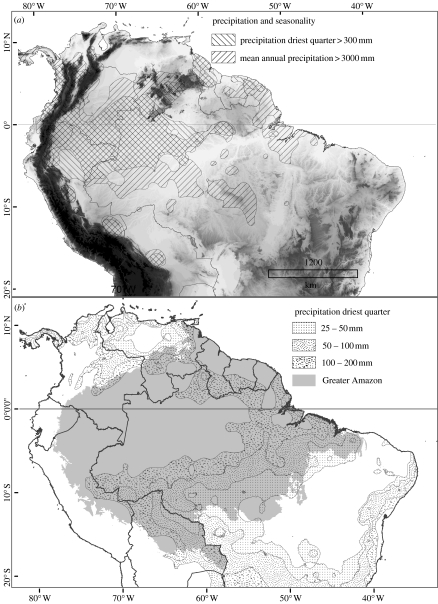
(*a*) Core Amazon is defined by high precipitation and low seasonality; extra-equatorial ‘wet spots’ are areas at higher latitudes which have these characteristics due to the interaction of topography and wind flows and (*b*) the areas at the greatest risk from future climate change are those in the southern and eastern Amazon with seasonal climate that could be shifted to a savannah-like climate.

We use datasets in the public domain and published information to identify regions and landscapes that will be resilient to climate change based on these assumptions. The WorldClim dataset (Hijmans *et al*. 2004) was used to characterize the precipitation regimes, and a version of the SRTM digital elevation model (Jarvis *et al*. 2006) was used to characterize topographic features. The GLC2000 map for South America was used to document land cover in the Amazon and Andes (Eva *et al*. 2002). We define the study area as the ‘greater Amazon’, which includes the Amazon basin in its entirety including the montane grasslands of the high Andes, as well as the Amazon wilderness area (Eva & Huber 2005), which includes other watersheds that share forest and savannah ecosystems similar to the principal ecosystems of the Amazon basin (figure 1).

## 3. Results and recommendations

### (a) Protect areas with stable high-precipitation regimes

If the risk from climate change is associated with reduced rainfall and increasing seasonality, then the areas with the highest actual rainfall and least seasonality, such as the central and western Amazon, are areas with the highest probability of avoiding the negative impacts from these changes. The stability of precipitation regimes in this region can be inferred from both historical reconstructions and climate models. Past climates oscillated between cool and dry during ‘glacial epochs’ versus warm and wet during ‘interglacial epochs’ (Mayle *et al*. 2004); future climates will be warmer and several GCMs predict that the Amazon will be drier (Betts *et al*. 2004; Li *et al*. 2006). In spite of the differences between past and future climates, the geographical characteristics that kept the western Amazon wetter than peripheral regions are likely to keep the region wetter in almost any potential future scenario.

The Amazon receives most of its moisture from the evaporation of water vapour over the Atlantic Ocean, which is transported into the Amazon by the east–west trade winds that are a result of planetary rotation and proximity to the equator, an attribute that is inherently stable. In the central Amazon, convective circulation keeps rainfall levels high and the Andean Cordillera functions as a barrier to the westward flow of humid air forcing high precipitation in the western Amazon. The reduced precipitation and increased seasonality at higher latitudes are linked to the seasonal shift of the intertropical convergence zone (ITCZ) and associated monsoonal precipitation over the South American continent (Marengo 2006). Most models predict increasing dryness over the southern Amazon, as well as in a north–south corridor in the eastern Amazon (figure 1*b*). The cause of this corridor is linked to rain squalls that originate in the afternoon over the Atlantic, move westerly with the prevailing trade winds and pass over this region during the night when convection is suppressed due to cooling land surface temperatures (Malhi *et al*. 2008).

In defining the geographical limits of this high-precipitation region, here defined as the ‘Core Amazon’, we use two bioclimatic envelopes: mean annual precipitation greater than 2500 mm and the mean precipitation of the driest quarter greater than 300 cm. Like most stratifications, this is an approximation based on the arbitrary selection of transition points; consequently, two parameters are used to establish both expansive and restrictive definitions (figure 1*a*). This stratification identifies a number of areas situated at slightly higher latitudes that meet the criteria of both high precipitation and low seasonality. Studies in Peru and Bolivia have identified high-precipitation areas that occur where prevailing winds intersect mountainous topography. Regional wind flows are linked to continental weather phenomena such as the Atlantic trade winds (Guianas and Venezuela), the South American low-level jet (Peru and Bolivia) and winds associated with the Eastern Equatorial Counter Current (Chocó, Colombia). The floras of these ‘extra-equatorial wet spots’ have been shown to be more similar when compared to other areas at the same latitude (Killeen *et al*. 2007).

### (b) Exploit ecotones as a buffer for climate change

The term ecotone is used to describe a transition between two ecosystems at the local scale or between two biomes at the continental scale; both concepts provide opportunities for mitigating the impact of climate change on biodiversity. At the continental scale, populations of a species considered to be characteristic of a particular biome propagate under conditions that are within the climatic extremes for that species; however, populations near the ecotone may have genetic traits distinct from core populations pre-adapting them to the physiological stress of climate change. The importance of climate in establishing the limits among biomes has led some biogeographers to refer to continental-scale ecotones as climatic transition zones (Ab'Saber 1977). The landscapes within these areas are characterized by habitat mosaics that reflect differences in soil humidity, texture, depth to bedrock and water table and geological substrate. These mosaics are occupied by species assembled in communities that reflect the presence of micro-environmental constraints in an area where climate stress is the overriding macro-environmental characteristic.

In geologically diverse and topographically variable landscapes, ecotone complexity leads to high levels of biodiversity (Killeen *et al*. 2002). However, unlike the Core Amazon where alpha diversity (species richness) makes the predominant contribution to overall (gamma) diversity, climatic transition zones are characterized by high levels of beta diversity (habitat richness). Evidence from palaeoecological studies show that some landscapes in the climatic transition zones are composed of hundreds (thousands) of micro-refugia that have persisted over millennia (Killeen *et al*. 2002; Mayle *et al*. 2004). Ecotones have been hypothesized to have hosted unique evolutionary processes that have contributed to the accumulation of tropical biodiversity (Smith *et al*. 1997). Each habitat is characterized by a suite of species and other specialized species exist on the ecotones among habitats. Where landscapes are spatially complex, there is a concomitant multiplication of ecotone types, leading to niche multiplication and opportunities for the evolution of new species (Moritz *et al*. 2000).

The long debate on the dynamic between forest and savannah interfaces is the product of this complex relationship between climate and substrate. The intensity of seasonality establishes the limit to the distribution of dominant Amazonian tree species, but the geographical breadth of the forest—savannah transition zone is a testament to the importance of edaphic constraints (Furley *et al*. 1992; Solórzano 1999).

Today's ecotones provide lessons for managing Amazonian landscapes that might become drier in the future, particularly those landscapes covered by seasonal moist forest in the southern and eastern Amazon. Botanical research has shown that the Amazon flora has strong regional differentiation (Daly & Mitchell 2000) and that plant communities vary over latitudinal and longitudinal gradients (ter Steege *et al*. 2006). Less well documented is the variability associated with geology, topographic relief, soil texture and fertility. The geomorphological variability and the subsequent beta diversity in the Amazon lowlands are not only a potential asset that can be used to mitigate the impacts of climate change but are also a strategically important component of regional biodiversity (Condit *et al*. 2002).

Fortunately, there is a considerable topographic variability within climatic transition zones and the adjacent areas with the highest probability of being impacted by the climate change (figure 1). Unfortunately, this is also the sector of the Amazon that is at most risk from mining, non-sustainable logging, agriculture and ranching; consequently, it will feel the compound impact of land-use change and increased drought. Knowing how the spatial configuration of habitats and the structural features of landscapes can mitigate climate change should be used to establish conservation priorities at the local, regional and continental scales.

### (c) Incorporate environmental gradients in conservation corridors

The main rationale for creating conservation corridors has been to mitigate the impact of habitat fragmentation, avoid local extinctions events and prevent the genetic deterioration of populations; however, it is now recognized that they may also help to mitigate the impact of climate change. Conservation corridors should be characterized by environmental gradients with sufficient natural habitat to create land-use mosaics that will maintain functional habitat connectivity. The gradients can be broad and diffuse or short and sharp, and both situations present risks and opportunities for conservation. Sharp gradients may allow species to shift ranges over relatively short geographical distances, while populations that exist across diffuse gradients may have the genetic plasticity that allow them to adapt to the climate change. Identifying the environmental constraint that regulates a gradient is vitally important for designing a management strategy. For example, the sharp gradients may reflect disturbance (fire), an abrupt change in the geological substrate or rain shadow; each situation will require different management practices to allow species to make successful shifts in range distributions across these gradients.

Altitude and latitude are two obvious large-scale gradients but are compounded by other smaller-scale environmental factors. The latitudinal gradient is essentially a precipitation gradient, but it is compounded by other gradients that are correlated with longitude, including soil fertility and geomorphology. The southeast Amazon will be more seriously stressed when compared with the southwest Amazon where proximity to the Andes will maintain precipitation near current levels (Li *et al*. 2006). Soils tend to be more fertile and have a higher sand fraction near to the Andes and the landscape is punctuated by geological features that amplify the impact of precipitation (Killeen *et al*. 2007). Similar climatic and edaphic interactions exist on the lowland landscapes in the northern Amazon, but are further complicated by weather patterns that originate over the Pacific Ocean and the Caribbean, as well as the topographic variability of the Guyana shield region. The importance of climate and soil interactions constrains not only the transitions between savannah and forest but also among dry and humid forests types (Pennington *et al*. 2004; Killeen *et al*. 2006), as well as among the multiple different types of Cerrado shrub and grassland variants (Solórzano 1999; Ratter *et al*. 2006).

Montane species distributions reflect multiple gradients including temperature, radiation, orographic rain, cloud density, slope, aspect and disturbance, all of which are directly or indirectly correlated with elevation (Kessler 2000). Although mean temperature gradients will change, extreme events such as frost are probably more important in limiting the upslope distribution of forest species; consequently, the montane habitats in southern Peru and Bolivia might experience more pronounced upward shift in species distributions as southern cold fronts decrease in frequency and intensity than similar elevations in Ecuador and Colombia.

Of particular concern is the potential impact of climate change on cloud bases (Pounds *et al*. 1999; Still *et al*. 1999), because cloud impacted forests and grasslands, known as *páramo* in the northern Andes, are highly diverse and rich in endemic species. However, the cloud forests of the eastern Andes may be more resilient than models predict, because the frequency of cloud cover is the result of complex interactions among local topographic features, prevailing winds and diurnal air flows (Killeen *et al*. 2007). The montane gradients are influenced by human disturbance, particularly at the ecotone between cloud forest and grasslands where fire may constrain the upward distribution of cloud forest species. In the central Andes, semi-arid habitats (*puna*) are situated above the humid grasslands and eventually give way to rock fields where availability of water due to prolonged freezing limits the growth of bryophytes and grasses.

### (d) Mitigate the impacts from transportation corridors

Infrastructure development is a priority investment by governments throughout South America and transportation projects are planned that will radically change the Amazon. Transcontinental highways will transect the southern Amazon and traverse the Andean piedmont from southern Peru to Colombia, while two parallel corridors will connect the central Amazon with the Caribbean. Hydrocarbon exploration is undergoing rapid expansion in the Core Amazon, where pipelines will open remote regions to migration and settlement. The expansion of these transportation corridors will be accompanied by a proliferation of secondary roads that will lead to broad deforestation belts that will effectively fragment the Amazon into large isolated forest blocks and impede the ability of species to shift ranges or persist long enough to adapt physiologically to climate change. Furthermore, the resultant increased production in agricultural, biofuel and mineral commodities will require the creation of waterways that will alter and fragment aquatic ecosystems. The existing environmental evaluation methodologies do not evaluate the impact of infrastructure investments on global warming, such as the impact of deforestation on greenhouse gas emissions. Although most require an evaluation of conservation strategies, they do not consider how conservation corridors can mitigate the compound and synergistic impacts from large-scale fragmentation, habitat reduction and climate change on species distributions (Killeen 2007).

## 4. Discussion and geographical synthesis

### (a) Protect the Core Amazon

The protection of the Core Amazon should be the foundation of all regional conservation strategies regardless of the dimensions of climate change. The western and central Amazon has the highest measured levels of *alpha* diversity on the continent (ter Steege *et al*. 2006), a phenomenon that conforms to a global trend where species diversity is correlated with latitude. There are several hypotheses that explain this phenomenon, all of which are relevant for the Amazon: (i) the existence of a large geographical area, (ii) the maximum potential energy and abundant precipitation that characterize equatorial regions, and (iii) a stable climate over millennia. Current macro-ecological theory holds that this combination of biophysical attributes is responsible for the evolution and persistence of the extremely high species diversity in equatorial regions (Whittaker *et al*. 2001). The unique evolutionary history of the Core Amazon makes its conservation a global priority of the first order.

Unfortunately, the western edge of the Core Amazon has been heavily impacted by peasant agriculture that followed oil exploration and production in the 1960s and 1970s in both Ecuador and Colombia. Most of the rest of the Core Amazon remains wilderness and the opportunity costs for implementing policies to ensure its conservation are still relatively low. Although high rainfall areas have long been considered to be inappropriate for industrialized agriculture, the area is ideally suited for oil palm, a major feedstock for biofuels, and large-scale plantations exist in Ecuador and Colombia.

A similar logic identifies the extra-equatorial wet spots as conservation priorities; their current conservation ranges from relatively pristine (Guianas and Venezuelan Guayana) to areas subject to extreme human pressure (Chapare, Bolivia; Putumayo, Colombia; and Huallaga Valley, Peru). The importance of these areas as potential long-term future refugia is essentially unknown within these countries and is only beginning to be appreciated by international conservation organizations.

### (b) River corridors in the southern Amazon

**Figure 2:**
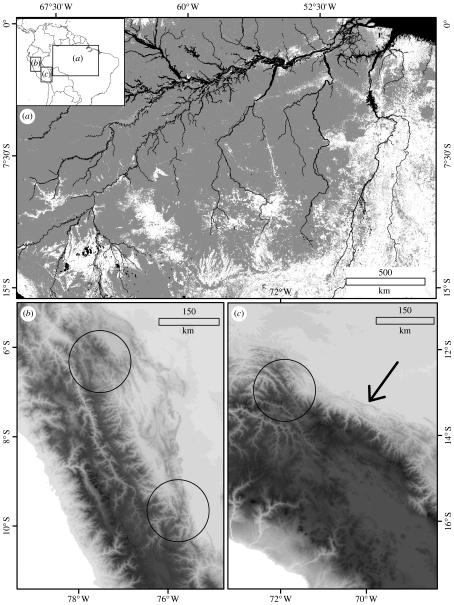
(*a*) The tributaries to the Amazon span the latitudinal gradient from the humid equator to seasonal habitats on the climatic transition zone of the southern Amazon and could form the basis of conservation corridors for both the aquatic and terrestrial ecosystems, (*b*) altitudinal corridors in the Andes should incorporate strategic areas where the front ranges are connected to the higher cordilleras (circles) or (*c*) areas where there is an uninterrupted transect between the lowlands and the high Andes (arrow).

There is a tendency within the conservation organizations to focus on terrestrial ecosystems even though it is recognized that aquatic systems are subjected to similar or even greater threats. Reductions in water flow from increased drought and seasonality will have large negative impacts, irrespective of whether they arise from a climate-induced forest dieback or deforestation. Consequently, conservation strategies organized at the basin scale bring double dividends in the form of conservation of both the aquatic and terrestrial ecosystems. Riparian corridors in the southern Amazon offer a unique opportunity to leverage terrestrial and aquatic conservation investments in the context of climate change. Humid valley bottoms will provide refuge for humid forest species as the region becomes more seasonally drought stressed, while also serving as latitudinal corridors to allow species to migrate, shift ranges and eventually adapt to future climate change. Riparian corridors can be used to create a skeleton that will conserve the essential connectivity of the historical Amazon in even the most radically changed future climate scenario (figure 2*a*).

Ironically, one of the measures to reduce global warming, the increased use of hydroelectric energy, is a threat to Amazonian aquatic biodiversity. Energy utilities prefer to place a single large hydro facility near the bottom of a river basin to maximize energy production, but that dam will cause more damage to migratory phenomena than dozens of smaller facilities situated on secondary and tertiary tributaries (Goulding *et al*. 2003). If a large watershed is to be modified due to economic expediency, then future investments should be concentrated on that same watershed in order to extend the life of the keystone energy facility by reducing sedimentation (e.g. Araguaia–Tocantins) and conserve the migratory populations in other watersheds (e.g. Xingu and Madeira). The development and modification of some watersheds is inevitable, but that should be mitigated by a commitment to keep some river basins entirely free of development.

### (c) Altitudinal corridors in the Andes

While change in precipitation regimes will largely be expressed on the latitudinal gradient and lowland rainforest, increases in temperature will impact the elevation gradient that defines montane habitats. In those parts of the Andes where the tops of mountain are above the permanent frost line, there is potential space for the upward displacement of species as a form of adaptation. An ideal corridor would incorporate an uninterrupted sequence of natural habitat stretching from lowland forests to the ice fields of high cordilleras. This may appear to be a straightforward elevation gradient, but it has several bottlenecks to plant and animal migration. The first are the human settlement zones on the Andean piedmont, particularly in the Putumayo of Colombia, Amazonian Ecuador, the *Selva Central* in Peru and the Chapare region of Bolivia, as well as incipient colonization zones in Loreto and Madre de Dios provinces in Peru and Iturralde Province, Bolivia. Similarly, many of the humid valleys in the front ranges have been long occupied by human populations, because tropical crops thrive and human health issues from tropical disease are minimal at these elevations.

A non-human-related bottleneck is situated at the crest of the front ranges, many of which are isolated from the main cordillera by deep valleys with contrasting drier climates (Killeen *et al*. 2007). These ridges may lack sufficient area and vertical relief to accommodate species that must shift their distribution upslope in response to raising temperatures or cloud bases (Pounds *et al*. 1999; Bush *et al*. 2004). Some of these ridges come together at selected points in the eastern Andes to form bridge-like connections with higher elevations and these areas should be identified as conservation priorities (figure 2*b*). Similarly, there are sectors in southern Peru and Ecuador that lack these intervening valleys and offer unique opportunities to create uninterrupted altitudinal gradients from the piedmont to the high Andes (figure 2*c*).

Another serious bottleneck on the flanks of the eastern Cordillera is the abrupt treeline that separates the cloud forest from humid grasslands. This ecotone has been hypothesized to result from fire used as a management tool by traditional pastoralists and cloud forests species would grow at higher elevations in the absence of human populations. There is concern that the treeline will stay locked in position and impede species that must adapt to climate change by the displacement of their altitudinal range distribution (Bush *et al*. 2004). The severity of this bottleneck needs to be better documented and mitigation measures developed to convince traditional pastoralists to refrain from using fire as a management tool. One approach would be to generate carbon credits from the afforestation programme of the clean development mechanism to compensate communities for changing their land-use practices.

The Andes represent a major potential asset in the search for strategies that can mitigate the impact from climate change, but their topographic complexity presents challenges that require strategic plans to ensure that natural habitats and ecological processes at key bottlenecks are protected to ensure habitat connectivity.

## 5. Conclusions

The impact of climate change in the Amazon is uncertain. The actions needed to manage the risks associated with that uncertainty bring additional benefits to conservation regardless of the direction, magnitude or the regional eccentricity of those changes. Most of the actions need to be taken sooner rather than later, because globalization and regional integration of development plans is bringing accelerated change to the Amazon. Species can adapt to climate change—they have done so in the past and can do so in the future—but they need to be provided with the conditions and time that allow them to adapt.
